# Refractory Genital HPV Infection and Adult-Onset Still Disease: A Case Report and Literature Review

**DOI:** 10.1097/MD.0000000000003169

**Published:** 2016-04-18

**Authors:** Xin Yu, Heyi Zheng

**Affiliations:** From the Department of Dermatology, Peking Union Medical College Hospital, Chinese Academy of Medical Sciences and Peking Union Medical College, Beijing, China.

## Abstract

Adult-onset Still disease (AOSD) is a systemic autoimmune disease (AIID) that can develop after exposure to infectious agents. Genital human papillomavirus (HPV) infection has been reported to induce or exacerbate AIIDs, such as systemic lupus erythematosus (SLE). No guidelines are available for the management of genital warts in AOSD.

Case report and literature review.

We report a patient who was diagnosed AOSD in the setting of refractory and recurrent genital HPV infection, demonstrating a possible link between HPV infection and AOSD. In addition, we also discuss the management of genital warts in patients with AOSD.

To the best of our knowledge, no previous cases of AOSD with genital HPV infection have been reported in literature. We then conclude that the patient AOSD may be triggered by primary HPV infection. Larger number of patient samples is needed to confirm whether HPV could trigger AOSD.

## INTRODUCTION

Condyloma accumulata (CA) or anogenital wart is a common sexually transmitted disease in China characterized by a papillomatous tumor in the anogenital area.^1^ It is caused by human papillomavirus (HPV) infection, commonly seen genotypes 6 and 11. Previous studies have showed that HPV infection or HPV vaccine might trigger systemic lupus erythematosus (SLE).^2^ An increased genital HPV infection was observed in women with SLE (12–20% vs 7%).^3,4^ Recurrences of HPV infection are common in immunocompromised population. Moreover, genital warts in those populations demonstrate a higher rate of malignant transformation.^5^ However, no guidelines are available for the treatment of genital warts in autoimmune inflammatory diseases (AIIDs). Treatment of genital warts in AIID is difficult since it is refractory to standard treatment.

Adult-onset Still disease (AOSD) is an uncommon systemic autoimmune inflammatory disorder, with an estimated prevalence lower than 1/100,000.^6^ It affects mainly young adults and typically manifests as fever of unknown origin (FUO).^7^ Other manifestations include arthralgia or arthritis, and transient maculopapular rash, sore throat, lymphadenopathy, hepatosplenomegaly, and elevated serum inflammatory markers and antinuclear antibodies. The cause and pathogenesis of AOSD is unknown. Infectious agents such as rubella, cytomegalovirus (CMV), Epstein–Barr virus (EBV), hepatitis virus, *Chlamydia pneumoniae*, *Yersinia enterocolitica* 3 and 9, *Brucella abortus*, and *Borrelia burgdoferi* have all been implicated as triggers in AOSD.^8^ Moreover, vaccination was also implicated in the initiation and immune reaction of AOSD. However, as far as concerned, no literature has reported the association between genital HPV infections and AOSD.

Here, we report a case of AOSD following a refractory genital HPV infection. This case suggests a causal relationship between genital HPV infection and development of AOSD. In addition, we also discuss the management of genital warts in patients with AOSD.

### Consent

Written informed consent was obtained from the patients for publication of this case report and any accompanying images. A copy of the written consent is available for review by the editor of this journal.

## CASE REPORT

**FIGURE 1:**
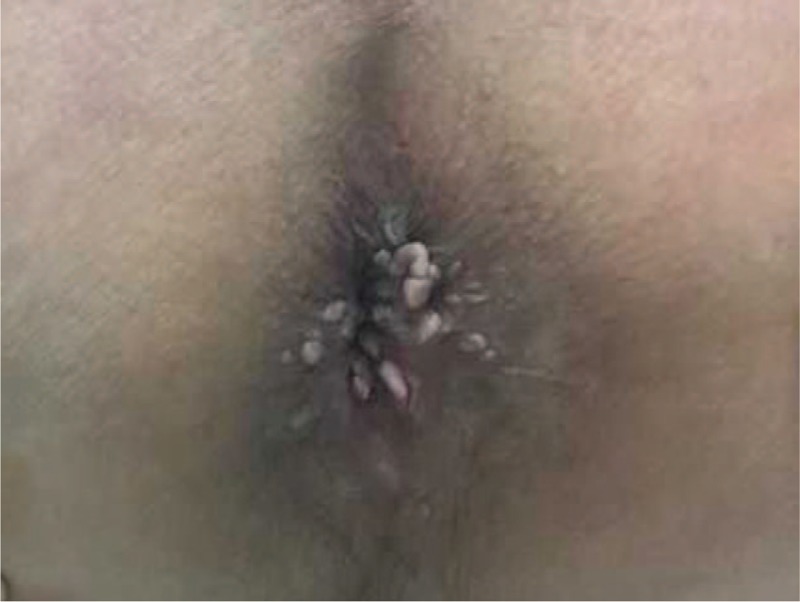
Twenty molluscoid lesions of 1 to 5 mm in diameter and widespread papillary and cauliflower-like condyloma acuminate.

A 35-year-old man presented to our dermatology outpatient clinic with a 1-year history of multiple perianal warts and 6-month history of AOSD in October 2015. He denied any unprotected anal sex with other partners. Cutaneous examination demonstrated that about 20 molluscoid lesions of 1 to 5 mm in diameter and widespread papillary and cauliflower-like condyloma acuminata (Figure 1) spread on the perianal region without any symptoms. He was started on weekly liquid nitrogen therapy for 7 months from October 2014 to April 2015. However, his perianal warts were still recalcitrant. On March 2015, he developed intermittent fevers, polyarthralgias, and maculopapular rash and admitted to the emergency department.

**FIGURE 2:**
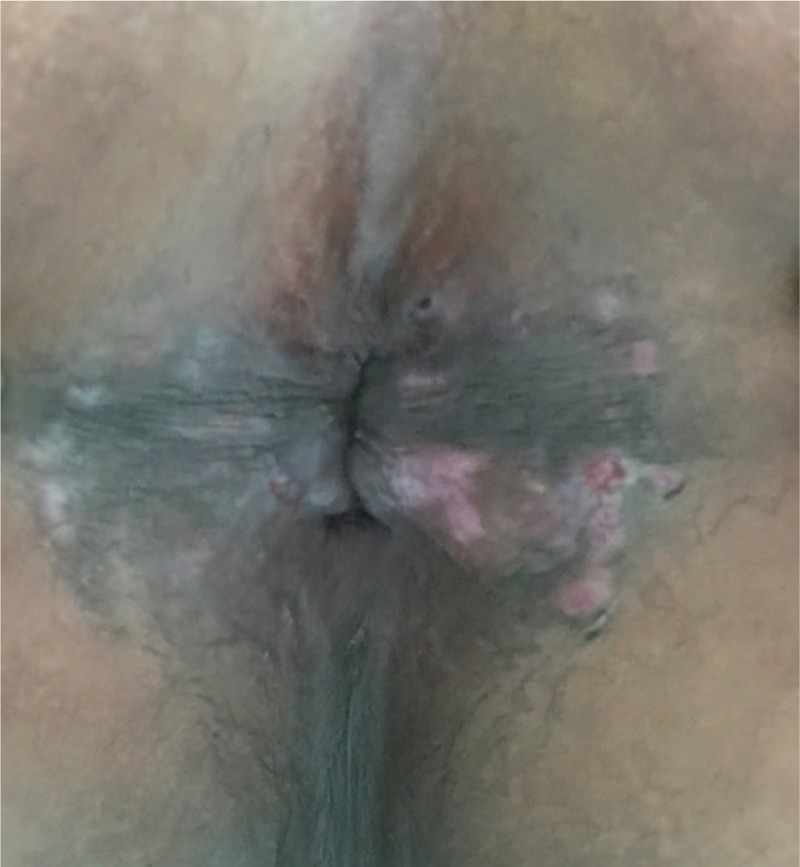
Anal lesions after treatment. The condyloma acuminate was much smaller and limited.

Workup to investigate the cause of fevers and polyarthralgias was as follows: serology for hepatitis viruses A, B, C, D, E, EBV, herpes simplex virus (HSV), human immunodeficiency virus (HIV), CMV, toxoplasmosis, and brucellosis. All tests were negative for acute or active infection. His lab examinations were negative for antinuclear antibodies (ANA) and rheumatoid arthritis autoimmune antibody spectrum. After excluding the possibilities of infection, neoplasms, and other AIIDs, diagnosis of AOSD was considered by rheumatologist according to Yamaguchi criteria. He received prednisone (PSL) 30 mg/day and MTX 15 mg/qw. His symptoms of fever and arthralgia gradually resolved, without recurrence of the (AOSD)-like manifestations following initiation of therapy. However, continued eruptions of genital lesions with increased severity occurred even with weekly cryotherapy. In May 2015, he received 5-aminolevulinic acid (ALA)-mediated PDT (ALA-PDT) treatment and stopped the treatment with cryotherapy. However, his anal warts were still recurrent with increased number and larger lesions than before. He came to our department to seek for further and better therapeutic strategy. Combined therapy using cryotherapy plus ALA-PDT (topical photodynamic therapy (PDT) using aminolevulinic acid) was initiated. His anal lesions gradually disappeared (Figure 2). He was appreciated and satisfied with the treatment.

## DISCUSSION

### Genital HPV Infection and Adult-Onset Still Diseases

HPV may be transmitted via 3 methods: sexual transmission, vertical transmission, or extragenital contact.^9^ There was no evidence of sexual transmission or any other sexually transmitted disease in this case. Therefore, extragenital contact appears to be the most plausible explanation. Moreover, a detailed history taking confirmed that HPV infection was before the development of AOSD in this case. The etiology of AOSD remains unknown.

AOSD is a diagnosis of exclusion when infections, neoplastic etiology, and AIIDs are excluded.^10^ But there is no published literature on AOSD after HPV infection presenting as genital warts. The development of AOSD following genital HPV infection indicates that HPV infection might be the trigger of AOSD. The role of infection has been extensively studied to investigate the possible etiologies of AOSD. Agents such as rubella, CMV, EBV, hepatitis virus, *C pneumoniae*, *Y enterocolitica* 3 and 9, *B. abortus*, and *B. burgdoferi* have all been implicated as triggers in the pathogenesis of AOSD. The underlying mechanisms are complicated and unknown. Also, patients with AOSD had a higher rate of HPV infection because of the immunocompromised condition induced by glucocorticoid and immunosuppressive drugs used.^11^ AOSD has been reported to be associated with high ferritin levels. Previous report showed that hyperferritinemic syndrome might be triggered by virus infection. Therefore, high ferritin levels after HPV infection might be the potential underlying mechanism of these 2 diseases.^12^

### Genital HPV Infection in Autoimmune Inflammatory Diseases

Infectious agents have been proved to induce or exaggerate AIIDs. In addition, vaccines have been reported to induce AIIDs. Genital HPV infection has been associated with SLE. Patients with a recent SLE diagnosis had disturbingly increased levels of HPV infections.^13^ Genital HPV infection was higher in SLE women than in the general population (12–20% vs 7%).^4,14^ In addition, an increased prevalence of abnormal Pap smears was reported in women with systemic sclerosis compared to the general population (25.4% vs 13.8%).^15^ More importantly, a case of psoriasis triggered by genital warts (HPV infection) was reported recently.^16^ However, in patients with rheumatoid arthritis and Sjögren's syndrome (SS), Pap smear revealed no significant differences in HPV status compared to controls.^17^ There are no epidemiological data on genital HPV infection in other AIIDs such as psoriasis and vasculitis. Moreover, the use of immunosuppressive treatments in AIID can induce genital HPV infection. Disseminated genital HPV infection may happen in glucocorticoid-treated patients.^18^ A significantly increased risk of cervical intraepithelial neoplasia (CIN) was observed in women with SLE given intravenous cyclophosphamide with prednisone or azathioprine compared to those given prednisone alone or with azathioprine.^19^

### Management of Genital HPV Lesions in Patients With Adult-Onset Still Diseases

Recurrences of HPV infection are common in immunocompromised population. HPV infection is refractory to standard treatment in those populations. In addition, it has a higher incidence of malignant transformation, substantially affecting the patient's quality of life. Therefore, it is crucial to control the HPV infection to prevent malignant transformation. However, no guidelines are available about the prevention and treatment of genital HPV infection in patients with AOSD. Immunosuppressive or biological agents should not be discontinued during genital HPV infections. However, dermatologists may learn experience from genital warts in HIV-infected patients. HPV screening and prophylactic HPV immunization is recommended in patients with AIID and immunosuppressive therapy.

## CONCLUSIONS

Though uncommon, this case raises the question of whether genital HPV infection can trigger AOSD. Larger sample investigations are needed to confirm whether HPV could trigger AOSD.

## References

[1] Steben M Garland SM Genital warts. Best practice & research. *Clin Obstet Gynaecol* 2014; 28:1063–1073. 10.1016/j.bpobgyn.2014.07.002 25155525

[2] Soldevilla HF Briones SF Navarra SV Systemic lupus erythematosus following HPV immunization or infection? *Lupus* 2012; 21:158–161. 22235047 10.1177/0961203311429556

[3] Yu SL Chan PK Wong CK Antagonist-mediated down-regulation of Toll-like receptors increases the prevalence of human papillomavirus infection in systemic lupus erythematosus. *Arthritis Res Ther* 2012; 14:R80. 22513098 10.1186/ar3803 PMC3446454

[4] Klumb EM Pinto AC Jesus GR Are women with lupus at higher risk of HPV infection? *Lupus* 2010; 19:1485–1491. 20605875 10.1177/0961203310372952

[5] Rodriguez AC Schiffman M Herrero R Rapid clearance of human papillomavirus and implications for clinical focus on persistent infections. *J Natl Cancer Inst* 2008; 100:513–517. 18364507 10.1093/jnci/djn044 PMC3705579

[6] Jamilloux Y Gerfaud-Valentin M Henry T Treatment of adult-onset Still's disease: a review. *Ther Clinical Risk Manag* 2015; 11:33–43. 25653531 10.2147/TCRM.S64951 PMC4278737

[7] Efthimiou P Paik PK Bielory L Diagnosis and management of adult onset Still's disease. *Ann Rheum Dis* 2006; 65:564–572. 16219707 10.1136/ard.2005.042143 PMC1798146

[8] Efthimiou P Georgy S Pathogenesis and management of adult-onset Still's disease. *Semin Arthritis Rheum* 2006; 36:144–152. 16949136 10.1016/j.semarthrit.2006.07.001

[9] Gavillon N Vervaet H Derniaux E How did I contract human papillomavirus (HPV)? *Gynecol Obstet Fertil* 2010; 38:199–204. 20189438 10.1016/j.gyobfe.2010.01.003

[10] Fautrel B Adult-onset Still disease. Best practice & research. *Clin Rheumatol* 2008; 22:773–792. 10.1016/j.berh.2008.08.006 19028363

[11] Paternoster DM Cester M Resente C Human papilloma virus infection and cervical intraepithelial neoplasia in transplanted patients. *Transplant Proc* 2008; 40:1877–1880. 18675077 10.1016/j.transproceed.2008.05.074

[12] Betancur JF Navarro EP Echeverry A Hyperferritinemic syndrome: Still's disease and catastrophic antiphospholipid syndrome triggered by fulminant Chikungunya infection: a case report of two patients. *Clin Rheumatol* 2015; 34:1989–1992. 26233722 10.1007/s10067-015-3040-9

[13] Nath R Mant C Luxton J High risk of human papillomavirus type 16 infections and of development of cervical squamous intraepithelial lesions in systemic lupus erythematosus patients. *Arthritis Rheum* 2007; 57:619–625. 17471531 10.1002/art.22667

[14] Dugue PA Lynge E Rebolj M Increased risk of high-grade squamous intraepithelial lesions in systemic lupus erythematosus: additional data from Denmark. *Autoimmun Rev* 2014; 13:1241–1242. 25151614 10.1016/j.autrev.2014.08.004

[15] Bernatsky S Hudson M Pope J Reports of abnormal cervical cancer screening tests in systemic sclerosis. *Rheumatology* 2009; 48:149–151. 19074957 10.1093/rheumatology/ken442

[16] Jain SP Gulhane S Pandey N Human papilloma virus infection and psoriasis: did human papilloma virus infection trigger psoriasis? *Indian J Sex Transm Dis* 2015; 36:201–203. 26692619 10.4103/2589-0557.167178 PMC4660567

[17] Cirpan T Guliyeva A Onder G Comparison of human papillomavirus testing and cervical cytology with colposcopic examination and biopsy in cervical cancer screening in a cohort of patients with Sjogren's syndrome. *Eur J Gynaecol Oncol* 2007; 28:302–306. 17713098

[18] Ko MJ Chu CY Disseminated human papillomavirus type 11 infection in a patient with pemphigus vulgaris: confirmed by DNA analysis. *J Am Acad Dermatol* 2004; 51 (5 Suppl.):S190–S193. 15577767 10.1016/j.jaad.2004.04.037

[19] Ognenovski VM Marder W Somers EC Increased incidence of cervical intraepithelial neoplasia in women with systemic lupus erythematosus treated with intravenous cyclophosphamide. *J Rheumatol* 2004; 31:1763–1767. 15338497

